# *Pseudomonas aeruginosa* in Chronic Lung Infections: How to Adapt Within the Host?

**DOI:** 10.3389/fimmu.2018.02416

**Published:** 2018-10-22

**Authors:** Emmanuel Faure, Kelly Kwong, Dao Nguyen

**Affiliations:** ^1^Department of Medicine, McGill University, Montreal, QC, Canada; ^2^Research Institute of the McGill University Health Center, Montreal, QC, Canada

**Keywords:** *Pseudomonas aeruginosa*, cystic fibrosis, immune evasion, chronic lung infection, host evasion, bacterial adaptation

## Abstract

Bacteria that readily adapt to different natural environments, can also exploit this versatility upon infection of the host to persist. *Pseudomonas aeruginosa*, a ubiquitous Gram-negative bacterium, is harmless to healthy individuals, and yet a formidable opportunistic pathogen in compromised hosts. When pathogenic, *P. aeruginosa* causes invasive and highly lethal disease in certain compromised hosts. In others, such as individuals with the genetic disease cystic fibrosis, this pathogen causes chronic lung infections which persist for decades. During chronic lung infections, *P. aeruginosa* adapts to the host environment by evolving toward a state of reduced bacterial invasiveness that favors bacterial persistence without causing overwhelming host injury. Host responses to chronic *P. aeruginosa* infections are complex and dynamic, ranging from vigorous activation of innate immune responses that are ineffective at eradicating the infecting bacteria, to relative host tolerance and dampened activation of host immunity. This review will examine how *P. aeruginosa* subverts host defenses and modulates immune and inflammatory responses during chronic infection. This dynamic interplay between host and pathogen is a major determinant in the pathogenesis of chronic *P. aeruginosa* lung infections.

## Introduction

Bacterial pathogens are most commonly studied for their ability to invade and injure the host, causing acute and invasive infections. In contrast, chronic infections present a distinct paradigm in infection pathogenesis which may challenge conventional notions of bacterial virulence and host defenses. To healthy individuals, *Pseudomonas aeruginosa* (PA) is a ubiquitous Gram-negative bacterium commonly encountered in the environment and readily cleared by host defenses. However, PA is also a formidable opportunistic pathogen that can cause invasive and fulminant infections, such as acute pneumonia or bloodstream infections, in immune compromised hosts. Remarkably, the same pathogen also causes chronic infections that persist for months to decades, such as the chronic lung infection in individuals with the genetic disease cystic fibrosis (CF). Chronic PA infections thus result from a dynamic and complex interplay between pathogen and host, where bacteria persist without causing overwhelming host injury, and where host defenses fail to eradicate the pathogen.

PA has a large genome (>6 Mb) that encodes many regulatory genes involved in sensing environmental signals, controlling expression of virulence factors, metabolism and resistance mechanisms. PA thus readily adapts to a wide range of environments and can exploit this versatility to enhance its long-term survival and persistence in the host. Importantly, host-pathogen interactions evolve over time and anatomical space, with the balance fluctuating between host recognition and vigorous activation of defense mechanisms, and immune evasion and tolerance by the host.

Chronic PA lung infections in individuals with CF persist for decades and provide a unique opportunity to examine how a bacterial pathogen can adapt to its host, modulate host responses and shift between different infection phenotypes. It is widely recognized that CF disease is associated with several intrinsic host defects, including impaired mucociliary clearance, and immune and inflammatory dysregulation. The implications of these host defects to the development of CF lung disease are beyond the scope of this review but may be found in excellent other ones (1–3). In this review, we will examine how PA defines the interactions central to the host immune and inflammatory response, and the bacterial adaptive strategies that promote bacterial persistence, and allow evasion and tolerance by the host during chronic infection. Specifically, we will highlight bacterial factors that undergo host-adaptation during chronic infections.

## Bacterial factors involved in host interactions and recognition

### Flagellin and flagellar motility

PA possesses a single polar flagellum composed of polymerized flagellin, its major structural protein, and attached to a transmembrane motor complex. The flagellar-host interaction plays a major role in defining the immune and inflammatory outcomes of PA infection, as the flagellar complex interacts with immune and non-immune cells through its structural components and as well as motility function.

The flagellar-host interactions have been extensively characterized at the cellular and molecular level. Flagellin is best known as a pathogen-associated molecular pattern that binds to the extracellular Toll like receptor TLR5 (4) and intracellular NOD-like receptor (NLR) neuronal apoptosis-inhibitory protein (NAIP) (5), in human (6), leading to activation of the pro-inflammatory MyD88 pathway and the NLRC4-inflammasome, respectively (7). TLR5 mediates a major component of the epithelial cytokine and chemokine responses leading to neutrophil recruitment in PA lung infection (8–10), and contributes to the production of pro-IL-1ß in monocytes and macrophages (11). Flagellin is also translocated by the Type-3 secretion system (T3SS) in the cytoplasm of mammalian cells, thereby activating the NAIP-NLRC4-inflammasome and inducing mature IL-1ß secretion (12, 13). Notably, IL-1ß promotes phagocytosis through its autocrine and paracrine effects (11, 14). Interestingly both flagellin and a motile flagellum are required to activate the NAIP-NLRC4-inflammasome (5, 15–17), but how host cells sense flagellar motility remains unclear. Beyond its ability to activate host cell signaling pathways, the flagellum also promotes adherence and colonization of host surfaces, and various specific targets have been identified including MUC1 mucin (18), heparin sulfate (19), surfactant protein A (20), and asialoGM1 (21).

During chronic infection, PA uses multiple strategies to evade flagellum-mediated host recognition. Flagellin expression is under the complex regulation by several global transcriptional regulators (22–25). It is repressed in mucoid variants which over-produce the exopolysaccharide alginate (26), during biofilm growth (27), upon as well as in response to the host nutritional and inflammatory environment. Notably, flagellin is repressed in the presence of CF sputum and airway fluid (28) as well as neutrophil elastase released at sites of inflammation (29). PA also expresses the secreted bacterial proteases AprA and LasB which cleave extracellular flagellin, suggesting an intrinsic mechanism to shut down flagellin-mediated immune recognition (30). Finally, loss of flagellar motility is common in host-adapted PA strains from CF lung infections and is associated with increased bacterial burden and disease severity (31). Genome sequencing studies of longitudinal PA strains have revealed evidence of convergent evolution and genetic mutations in regulatory genes such as *rpoN* and *fleQ* which lead to downregulation of flagellar expression and motility (32, 33). In fact, PA isolates recovered from chronic CF lung infections fail to activate the inflammasome due to reduced expression of flagellin and T3SS (34).

### Type 3 secretion system (T3SS)

The type III secretion system (T3SS) is a complex needle-like secretion machinery found in gram-negative bacteria that allows the translocation of bacterial effectors directly into the cytoplasm of host cells, causing cytotoxicity, or subversion of host defenses (35). The T3SS causes tissue injury, promotes bacterial dissemination and has been implicated in the pathogenesis of acute and invasive infections, including pneumonia (36–38). Four T3SS-dependent effectors have been identified in PA, namely ExoS, ExoT, ExoY, and ExoU, and have been recently reviewed elsewhere (35). The T3SS effectors cause disruption of host cell cytoskeleton (ExoS, T, and U) and cleavage of phospholipases (ExoU), leading to cell death, a breach of epithelial and endothelial barriers and killing of phagocytes (39–41). ExoS also dampens phagocytosis by interfering with lysosome signaling in macrophages (42, 43).

Beyond its role in cytotoxicity, the T3SS activates innate immune responses through secretion of IL-1ß (44). The T3SS apparatus itself, independently of exotoxin, can activate the NLRC4-inflammasome through NAIP recognition (44–46), leading to pyroptotic cell death and the secretion of mature IL-1ß and IL-18. Whether inflammasome activation contributes to the effective immune response to control bacteria, or to the immunopathology associated with PA lung infections remains incompletely understood. On one hand, inflammasome activation and IL-1R signaling may be protective at early stages of infection (47, 48). On the other hand, NLRC4 activation is associated with reduced alveolar macrophages, reduced PA clearance and increased neutrophil recruitment, leading to greater lung immunopathology and mortality in a murine model of acute lung infection (49, 50).

Chronic infections appear to select against T3SS-expressing PA. Although many CF patients carry antibodies against T3SS effector proteins (51), suggesting that these effector proteins were secreted at some stage of the infection, most PA strains isolated from chronic infection are T3SS-negative (34, 52, 53). Loss of T3SS results in dampened inflammasome activation and lesser pyroptotic cell death in macrophages and neutrophils (34). CF isolates are rarely ExoU+ (54), also consistent with the notion that acute cytotoxicity, particularly when conferred by ExoU, is less compatible with chronic infection. As discussed later in this review, several mechanisms contribute to the loss of T3SS in CF-adapted PA strains.

### Secreted proteases

PA produces several secreted proteases, which include LasB (also known as PA elastase or pseudolysin), LasA, AprA, and protease IV. Secreted PA proteases interact with a wide range of host molecules, leading to diverse outcomes, from degradation of structural components to modulation of inflammatory responses. The PA proteases are most studied for their ability to cause direct tissue damage, and they are primarily known as virulence factors involved in the pathogenesis of acute infections. LasB, a broad specificity metallo-protease, degrades elastin (55), disrupts epithelial tight-junctions (56), and reduce endothelial barrier integrity (57, 58). As a consequence, LasB mutants are attenuated in virulence in experimental models of bacteremia (59), acute pneumonia (60), or burn wound model (61).

PA proteases also alter host responses by degrading secreted mediators, leading to a dampening of inflammatory and immune responses, which likely contributes to its ability to evade host defenses. *In vitro* studies have shown that PA proteases potently degrades secreted mediators such as cytokines (e.g., INF-γ, IL-6), chemokines (e.g., IL-8/CXCL1, MCP-1, CXCL-5, RANTES/CCL5) (62–66), host defense components such as immunoglobulins (67, 68), antimicrobial peptides (e.g., LL-37) (69), and membrane receptors (e.g., protease-activated receptor PAR-1,2 and 4) (70, 71). LasB helps PA subvert alveolar macrophage activity by down-regulating the oxidative burst and production of complement factors (72). LasB mediated degradation of surfactant proteins SP-A and SP-D also leads to phagocytosis resistance (73, 74). Proteolysis of thrombin by LasB releases an anti-inflammatory thrombin-derived peptide FYT21, which inhibits the activation of the transcription factors NF-κB and AP-1 (75). Finally, AprA and LasB can degrade flagellin monomers, and thus blunt TLR5-mediated responses (30) and inflammasome activation (76). Interestingly, the inflammasome activation is also dampened due to proteolytic degradation of extracellular inflammasome components by PA proteases (76).

Although most PA isolates recovered from environmental sources or acute infections produce secreted proteases, protease-deficient PA isolates are commonly isolated from patients with CF and chronic obstructive pulmonary disease (COPD) chronically colonized with PA (77, 78). In fact, loss of secreted protease activity occurs as part of the genetic adaptation of PA to the host environment (see section below) and is associated with chronic and more advanced lung disease (32, 79). As secreted proteases dampen inflammation, loss of protease activity in CF-adapted PA variants conversely can promote exaggerated inflammation and lung immunopathology, as observed *in vitro, in vivo* in murine models of chronic PA lung infections and in CF patients (80). The impact of secreted PA proteases on host responses and pathology thus varies in different infection settings, such as acute vs. chronic, invasive vs. localized, as the presence or loss of proteases promote disease through different mechanisms of host interactions.

### Exopolysaccharides (EPS)

PA produces three extracellular polysaccharides (or exopolysaccharides), namely alginate, Psl, and Pel. They provide many protective properties and confer surface and self-adherence. They are constituents of the biofilm matrix, are involved in surface colonization and promote host immune evasion. A detailed review of these EPS and their distinct functions can be found elsewhere (81).

Mucoid PA overproduces the exopolysaccharide alginate and these strains are commonly associated with chronic CF lung infections and other chronic lung diseases (79, 82, 83). Alginate over-production (mucoidy) impairs host defenses and promotes bacterial persistence through several mechanisms. Alginate overproduction interferes with opsonophagocytosis and complement activation, scavenges ROS and inhibits phagocytic killing (82, 84, 85). It also confers resistance to host antimicrobials such as LL-37 and reactive oxygen species H_2_O_2_ (86). Whether mucoidy dampens host detection remains unclear. Mucoidy represses flagellar biosynthesis due to the co-regulation of flagellin and alginate (26), leading to reduced TLR5-dependent activation. However, mucoidy is linked with increases bacterial lipoproteins expression (87), which activates TLR2 in host airway epithelial cells (88), and is associated to greater resistance to the anti-inflammatory effects of corticosteroids (89).

Psl and Pel are exopolysaccharides which confer structural and aggregative properties to the biofilm matrix and contribute to the biofilm antibiotic tolerance (90, 91). Psl interferes with complement deposition and hinders neutrophil opsonophagocytosis and oxidative killing (92). Although its interactions with host cells are less well-characterized, Pel likely also contributes to resistance against neutrophil killing (93). PA genetic variants that overproduce Psl and/or Pel are found in chronic CF infections (94) and are associated with increased bacterial burden and host immune evasion (95).

### Lipopolysaccharides (LPS)

LPS (also known as endotoxin) is a major component of the outer membrane of Gram negative bacteria. LPS is composed of three components: the lipid A and core oligosaccharides that form the outer leaflet of the bacterial outer membrane, and the O-antigen polysaccharide which interacts with the extracellular environment. LPS is recognized by the Toll like receptor 4 and myeloid differentiation factor 2 complex (TLR4-MD2). The O-antigen consists of highly variable and immunogenic oligosaccharide repeats which elicit a strong humoral response (96).

During chronic infection, the LPS undergoes important adaptive changes at the level of its synthesis and structure, leading to modification of the lipid A structure and loss of O antigen which likely promote immune evasion. Lipid A acylation patterns or addition of positively charged components, renders the outer membrane more resistant to host antimicrobial peptides (97–99), modulates TLR4-MD2 receptor recognition and dampens host inflammation (100). PA isolates from chronic infection commonly express little or no O-antigen (101, 102). Mutations in LPS and O-antigen biosynthesis are common (32, 103, 104) and appear to be a hotspot of genetic variation and adaptation during chronic CF infections (105). Finally, O-antigen biosynthesis is also modulated by cyclic-di-GMP, a second messenger involved in the switch from motile to adherent lifestyle of PA (106). A summary of the bacterial factors/complex involved in the host adaptation during chronic PA infections is provided in Table 1.

**Table 1:** Bacterial factors/complex involved in host-adaptation during chronic PA infections.

**Bacterial factor/complex**	**Bacterial function**	**Host interactions**	**Adaptation in chronic infection**
Flagellum	- Macromolecular motility appendage which confers motility in low viscosity liquids through rotational movement - Flagellin is the principal structural component of the flagellar filament - Mediates biotic and abiotic surface adhesion	- Flagellin binds and activates TLR5 and intracellular Naip5 protein, leading to activation of MyD88 and NLRC4—dependent inflammatory pathways respectively - Promotes surface attachment and colonization by adhering to mucins, surfactant protein A, host surface molecules (e.g., heparin sulfate proteoglycans, AsialoGM1)	- Reduced flagellar motility and/or flagellin synthesis in response to mucin, neutrophil elastase and airway fluid, during biofilm growth, and due to genetic mutations in biogenesis or regulatory genes (e.g., *rpoN, fleQ*) - Dampened host recognition, phagocytic uptake and downstream activation of MyD88 and NLRC4—dependent pathways
Type IV pili (T4P)	- Macromolecular motility appendage which confers surface motility through extension, attachment, and retraction movement - Mediates sensing and adhesion to biotic and abiotic surfaces - Promotes biofilm formation (*in vitro*) - DNA uptake	- Binds host surface molecules (e.g., heparin sulfate proteoglycans and N-glycans) and promotes surface colonization - Promotes direct bacterial-host cell membrane contact and thus T3SS-dependent toxicity	- Reduced pilus-mediated motility due to regulatory control (e.g., cAMP and cyclic-di-GMP pathways) or due genetic mutations in biogenesis or regulatory genes - Reduced colonization and invasion of host tissues
Type 3 secretion system (T3SS)	- Needle-like structure that injects and translocates bacterial effector proteins across cellular membranes into the host cell cytoplasm	- Translocation of effectors proteins (ExoU, ExoY, ExoS, ExoT, flagellin) which interact with the eukaryotic cytoskeleton and immune responses in phagocytes and non-phagocytic cells - Translocation of flagellin and other flagellar components into host cytosol, leading to inflammasome activation	- Repressed expression due to regulatory control or mutations of regulatory genes (e.g., RetS/GacS, cyclic-di-GMP pathways) - Reduced host cell cytotoxicity and inflammasome activation
Type 6 secretion system (T6SS)	- Secretion/injection system that delivers effector proteins into prokaryotic and eukaryotic target cells - Involved in bacterial competition	- The effectors PldA and PldB activate the PI3K/Akt pathway, and VgrG2b interacts with microtubules, which promote bacterial internalization in non-phagocytic cells (*in vitro*)	- Expression potentially induced due to regulatory control or mutations of regulatory genes (e.g., RetS/GacS, cyclic-di-GMP pathways)
Exopolysaccharides	- Alginate scavenges reactive oxygen species and is overproduced in mucoid variants - Psl and Pel have aggregative properties that confer cell-cell and surface adherence - Major structural component of biofilm matrix, which contribute to biofilm antibiotic resistance	- Pel and Psl promotes adherence to host cell surface - Interferes with opsono-phagocytosis, phagocyte oxidative burst and killing	- EPS overproduction due to mutations or environment control in regulatory genes (e.g., *mucA*, cyclic-d-GMP pathway) - Co-regulation of EPS with other bacterial factors through common pathways (e.g., AlgT, cyclic-di-GMP) leads to repression of flagellar biosynthesis and T3SS activity, increased expression of bacterial lipoproteins (TLR2 agonists) in EPS over-expressing strains. - Impaired bacterial clearance
Lipolysaccharides (LPS)	- Lipid A component is embedded in the outer membrane - O-antigen is composed of highly variable oligosaccharide repeats exposed at the bacterial surface	- Lipid A binds TLR4-MD2 - O-antigen is a common antibody epitope - Confers resistance to complement killing and cationic antimicrobial peptides	- Different lipid A modifications with varying impact: enhanced or dampened TLR4 activation, leading to immune evasion or enhanced immune-stimulation - Loss of O-antigen due to mutations in biosynthetic genes, leading to immune evasion
Secreted proteases (LasA, LasB, AprA, Protease IV)	- Proteolytic degradation of extracellular peptides	- Degrades elastin, thrombin, fibrinogen, surfactant proteins A and D, complements proteins, immunoglobulins, cytokines, and other extracellular mediators - Degrades flagellin - Disrupts epithelial tight-junctions and reduces barrier integrity	- Loss of secreted protease activity due to genetic mutations in regulatory genes (e.g., LasR quorum sensing) - Reduced host tissue destruction and invasion - Dampened immune recognition - Increased accumulation of mediators and inflammation

## PA phenotypic and genetic adaption to host environments

During the process of chronic infection, PA adapts to the host environment and undergoes changes which promote bacterial survival and evasion of host defenses. Certain adaptive processes occur at the phenotypic and regulatory level, while others occur through genetic mutations and evolution. We will review here the key regulatory and genetic adaptive processes that PA undergoes during chronic PA infection.

### Biofilm lifestyle

In contrast to the free-living bacterial lifestyle termed planktonic, PA can also grow in a multicellular and sessile form, termed biofilms. Biofilms are formed by self-aggregated or surface-adherent bacteria encased within an extracellular matrix. Biofilms cause many chronic and non-invasive human infections such as medical device associated infections, chronic CF lung infection and chronic wound infections. Our understanding of *in vivo* host responses to PA biofilms is limited by the lack of animal infection models that mimic human biofilm infections. Our insights are thus primarily drawn from *in vitro* studies that examine the response of various cell types to biofilm bacteria. Biofilm formation and its role in disease pathogenesis have been the subject of recent reviews (81, 107), and only aspects relevant to host-biofilm interactions are outlined here.

Host responses to PA biofilms are complex, as biofilms may both stimulate or suppress the immune system. Biofilms may be less immune-stimulatory than their free-living planktonic counterparts. For example, the expression of flagellin and T3SS is down-regulated (108, 109), and the complement system is less activated (110) during biofilm growth. Furthermore, bacterial factors involved in host interactions may be embedded within the biofilm matrix and not readily accessible for host recognition. Conversely, biofilms can induce a robust neutrophilic response where neutrophils are activated, undergo oxidative burst and degranulate, but are immobilized (111–113). Biofilm PA can also trigger necrotic cell death in neutrophils (113), leading to further inflammation and collateral tissue damage.

Importantly, innate immune responses are less effective against biofilm than planktonic PA. As described above, exopolysaccharides constitute the major components of the biofilm matrix and contribute to biofilm resistance against host antimicrobials defenses and phagocytic killing. Biofilm infections are thus associated with a smoldering immune response that is ineffective at clearing bacteria but remains active enough to cause tissue damage over long periods of time.

### Regulatory control to switch bacterial lifestyle and infection strategy

PA is capable of phenotypically switching between its motile planktonic lifestyle and the sessile biofilm lifestyle through multiple and overlapping regulatory networks which include the RetS/GacS sensor pathway. Through the opposing functions of RetS and GacS and their signaling cascades, the RetS/GacS pathway converge on the regulator RsmA and is linked to the second messenger cyclic di-GMP. It coordinately controls the expression of motility, Pel and Psl exopolysaccharides, T3SS and Type VI secretion system (T6SS) -related gene (114, 115). Chronic infection is thus favored as PA represses its T3SS, motility and produces the exopolysaccharides that form the biofilm matrix. Interestingly, analysis of host-adapted PA strains from chronic CF infections identified genetic mutations in the RetS/GacS pathway, with the possibility that *retS* mutations promote a chronic infection state (116). Conversely, dysregulation of RetS/GacS pathway due to mutations in *gacS* or its regulator *ladS* can also cause excessive T3SS activity and cytotoxicity, leading to hyper-virulent PA strains that cause fulminant infections (117) or exacerbations during chronic CF infection (118).

Cyclic di-GMP is an intracellular bacterial secondary messenger that regulates multiple bacterial behaviors, most notably those involved in biofilm formation. The cellular level of c-di-GMP are modulated in response to environmental and intracellular signals, and affect expression of genes involved in flagellar and type IV pilus mediated motility, exopolysaccharide production and surface adhesion (115). Genetic variants that overproduce cyclic di-GMP display an auto-aggregative phenotype caused by the overproduction of Psl and Pel, have been recovered from chronic CF lung infections (94).

The RetS/GacS and sensor pathway, cyclic di-GMP signaling and other global regulators (e.g., quorum sensing, two component sensor regulators) allow PA to coordinately regulate numerous factors that define distinct bacterial infection strategies, namely acute and invasive disease, or chronic and localized disease. It is plausible that the ability of PA to phenotypically switch between acute and chronic virulence modes contributes to the complex disease phenotype it causes: the natural history of chronic PA lung infections is characterized by slowly progressive tissue pathology, but is also interrupted by periods of acute and more fulminant disease termed acute exacerbations. It is possible to speculate that exacerbation episodes may be caused in part by a phenotypic switch to acute virulence.

### Genetic adaptation during chronic infection

The bacterial genetic adaptation to host environments is a common theme during chronic infection. For PA, this has been best documented in chronic CF lung infection, and we suggest several excellent recent reviews (33, 116, 119) for a detailed discussion of the topic. In CF, factors that contribute to the mutagenesis of PA include the presence of hypermutator strains (120), and the pro-inflammatory environment of the CF lung rich in oxidative and nitrosative stresses (33).

During its long residence in the CF lung, PA populations show both genetic diversification as well as convergent evolution. On one hand, PA undergoes significant genetic and phenotypic diversification during chronic CF infection, a process likely attributable to the divergent evolution of clonally related PA inhabiting different regions and micro-environments of the lung (121). On the other hand, numerous studies have shown evidence of convergent evolution when comparing the PA genomes within patients over time, and across different patients (122). Genome sequence analyses show a strong positive selection for non-synonymous mutations in genes encoding or regulating virulence factors (e.g., T3SS, exotoxin A, quorum sensing), immunogenicity factors (e.g., O-antigen), motility (flagellar and T4P mediated motility), drug resistance (e.g., multidrug efflux pumps), and metabolism (e.g., iron uptake). Importantly, many of these mutations confer loss of function or secretion of extracellular factors (e.g., proteases, T3SS) and promote immune evasion (32, 123). For example, LasR quorum sensing and protease-deficient variants are observed in over a third of CF patients with chronic PA infections. This suggests that the host environment likely confers strong selective forces that shape host-pathogen interactions and drive the genetic adaptation of PA toward a state that promote bacterial survival and persistence in the face of host defenses.

## Advances and challenges in the development of alternative or adjuvant therapies for chronic PA infections

Alternative or adjuvant therapies that minimize direct bacterial damage to the host, that enhance protective host responses or subvert pathological ones, can improve infection outcomes (124). Such therapies are particularly needed in light of the alarming rise in drug resistance, and for drug tolerant chronic infections (125). The latter refers to the phenotypic state of slow growing and biofilm bacteria which are refractory to antibacterial killing even in the absence of drug resistance. Unfortunately, despite intense research efforts and many candidates in pre-clinical studies, the development of novel therapies in chronic PA infections has been arduous and met with very limited success so far.

Anti-virulence therapies target bacterial virulence without disrupting bacterial growth or viability. Although numerous PA targets (e.g., quorum sensing signaling, biofilm exopolysaccharides, T3SS complex, and effectors) and inhibitor molecules have been studied, very few have progressed past pre-clinical studies (126). Anti-virulence therapies face unique challenges due to the bacterial phenotypic heterogeneity and complex host interactions characteristic of chronic PA infections. First, many PA strains isolated from chronic infections do not express functional factors such as flagellum and T3SS, suggesting that these factors may not play as important a role in virulence during chronic infections as during acute PA infections. Furthermore, the genetic and phenotypic adaptation of PA to the host during chronic infection lead to extraordinary heterogeneity between different patients, as well as at different stages or anatomically distinct foci of disease within the same patient. Anti-virulence therapies may thus need to be tailored to specific patients and/or infection states (e.g., early infection or acute exacerbation) based on a more comprehensive microbiological profiling than currently available in the clinic.

Antibacterial antibodies can neutralize bacterial virulence factors, induce complement mediated lysis and enhance opsonophagocytic uptake and killing (127). Advances in antibody engineering and screening have accelerated antibody therapeutics, and a few anti-PA antibodies have reached clinical trials. Polyclonal anti-PA antibodies (PsAer-IgY) (128) are currently in Phase 3 clinical trials (NCT01455675) for the prevention of recurrent PA infections in CF patients. Monoclonal antibodies that target the exopolysaccharides alginate (AR-105, Aridis Pharmaceuticals) and Psl (129), the T3SS needle protein PcrV [MEDI3902, MedImmune (130); KB001 (131)], O11 serotype LPS [AR-101/KBPA101, Aridis Pharmaceuticals (132)], or combinations [e.g., bispecific anti Psl/PcrV MEDI3902, MedImmune (130)] are currently tested for the prevention or treatment of acute PA pneumonia but their utility in preventing or treating chronic infections remains to be determined (133).

Considering the intractable nature of chronic PA infection, an important strategy is also to prevent infection through approaches such as vaccine, antibody, enzyme or antibiotic-based treatments. Although several anti-PA vaccine targeting antigens such as LPS O-antigen, alginate, outer membrane or flagellar proteins showed promise in pre-clinical trials, their clinical efficacy in reducing the risk of chronic PA infection in susceptible individuals (such as CF patients) has been overall disappointing to date (134, 135).

## Conclusion

Chronic PA infection illustrates a paradigm of chronic bacterial infections where pathogens dampen host defenses, adapt and evolve within the host to persist. Understanding the pathogenesis of chronic PA infection thus requires an intricate assessment of bacteria, host responses, and their interactions over time. Host-PA interactions are exceptionally complex in chronic infections, as they involve numerous host cell types and bacterial factors. These interactions are further complicated by the common co-existence of other pathogens or polymicrobial communities that interact with both host and PA, and by the potential changes in the host due to factors such as aging or environmental exposures. While decades of research have provided us with vast mechanistic data on host-PA interactions, integrating these mechanistic insights into a whole system understanding of chronic infection and translating this knowledge into effective treatments remain a major challenge. The development of better *in vivo* models of chronic PA infection and tools to simultaneously probe host and pathogen over time is critical in order to gain a more integrated understanding of chronic infections.

## Author contributions

All authors listed have made a substantial, direct and intellectual contribution to the work, and approved it for publication.

### Conflict of interest statement

The authors declare that the research was conducted in the absence of any commercial or financial relationships that could be construed as a potential conflict of interest. The handling Editor declared a shared affiliation, though no other collaboration, with all of the authors KK, EF, and DN.

## Abbreviations

PA: Pseudomonas aeruginosa
CF: cystic fibrosis
cyclic di-GMP: cyclic diguanylate
EPS: exopolysaccharide
IL: interleukin
LPS: lipopolysaccharide
NAIP: neuronal apoptosis-inhibitory protein
ROS: reactive oxygen species
T3SS: Type-3 secretion system
T4P: Type 4 pili
TLR: Toll like receptor.
